# Restraint Stress Induced Hyperpermeability and Damage of the Blood-Brain Barrier in the Amygdala of Adult Rats

**DOI:** 10.3389/fnmol.2019.00032

**Published:** 2019-02-13

**Authors:** Guangming Xu, Yingmin Li, Chunling Ma, Chuan Wang, Zhaoling Sun, Yiwen Shen, Li Liu, Shujin Li, Xiaojing Zhang, Bin Cong

**Affiliations:** ^1^Hebei Key Laboratory of Forensic Medicine, Collaborative Innovation Center of Forensic Medical Molecular Identification, College of Forensic Medicine, Hebei Medical University, Shijiazhuang, China; ^2^Department of Forensic Medicine, School of Basic Medical Sciences, Fudan University, Shanghai, China; ^3^Forensic Science, Beijing Public Security Bureau, Beijing, China

**Keywords:** blood-brain barrier, restraint stress, tight junction, adherens junction, permeability, AQP-4, GLUT-1

## Abstract

Intense or prolonged exposure to stress can damage various brain structures, including the amygdala and hippocampus, which are associated with emotional-cognitive functions. Furthermore, this deterioration has been linked to a myriad of neurodegenerative and psychiatric disorders, in particular through disruption of the blood-brain barrier (BBB). However, insights remain scarce concerning the effects and mechanisms associated with stress on the BBB in the amygdala. This study explored the effects of restraint stress on the permeability and integrity of the BBB in the amygdala of male adult SD rats. Serum levels of corticosterone (CORT) and S100B were determined through ELISA. The permeability of the BBB was assessed by measuring Evans Blue (EB) leakage in tissue samples from the rats’ amygdala. These samples were immunostained for markers of tight junctions (Claudin-5, Occludin, ZO-1) and adherens junctions (VE-cadherin), as well as GLUT-1 and AQP-4. Staining was evaluated through confocal microscopy, and the level of expression of these proteins was quantified using the Western Blot (WB) technique. The ultrastructure of brain microvascular endothelial cells was assessed with transmission electron microscopy. Moreover, interleukin-1 beta (IL-1β) content in serum and amygdalar tissues were determined by employing ELISA. Exposure to restraint stress was associated with higher serum levels of S100B and EB leakage in amygdala tissues, especially in days 14 and 21 of the experiment, indicating increased permeability of the BBB. After restraint stress, significant decreases in protein expression were detected for tight junctions, adherens junctions and GLUT-1, while a significant increase was observed for AQP-4. The variation trends of fluorescence intensity generally paralleled these results. Following restraint stress, transmission electron microscopy ascertained enlarged gaps in tight junctions and thickened basal membranes in amygdalar capillaries. In addition, increased IL-1β contents in serum and amygdalar tissues were observed in the restraint-stressed groups. These findings suggest that restraint stress mediates time-dependent alterations in the permeability of the BBB, with modifications in the expression of proteins from tight junctions and adherens junctions, as well as ultrastructural changes in brain microvascular endothelial cells. And it was associated with the inflammation. These alterations may be associated with behavioral and cognitive dysfunctions and neurodegenerative disorders.

## Introduction

Stress is a non-specific psychobiological and neuroendocrine response experienced on encountering a threat. Although moderate stress plays an adaptive role and is necessary for survival, intense or prolonged exposure to stress can damage brain areas associated with emotional-cognitive functions—such as fear memory and anxiety–, including the amygdala and hippocampus (Sathyanesan et al., 2017; Lowery-Gionta et al., 2018). These changes may be involved in a variety of neurodegenerative and psychiatric disorders (Mcewen and Gianaros, 2010).

The amygdala is one of the most important nuclei in the limbic system, with an extremely broad array of connections and functions (Sah et al., 2003). It plays a critical role in emotional regulation concerning memories related to fear and anxiety (Tripathi et al., 2017). The amygdala appears to be particularly sensitive to stress-induced damage. Several studies have revealed restraint stress could induce atrophy of glutamatergic pyramidal neurons in the amygdala, potentially leading to mental disorders (Padival et al., 2013; Grillo et al., 2015). Our previous study has also suggested restraint stress to be linked with degeneration of amygdalar neurons and astrocytes in rats and induce behavioral changes, such as anxiety and depression (Dong et al., 2017). Nevertheless, the specific mechanisms underlying these phenomena remain unclear.

The blood-brain barrier (BBB) is a specialized and dynamic system, mainly consisting of brain microvascular endothelial cells (BMVEC), which interact directly with neurons, astrocytes, and pericytes (Tajes et al., 2014). BMVEC display high electrical resistance due to the expression of various proteins from tight junctions (TJ) and adherens junctions (AJ), such as occludin, ZO-1, claudin-5 and VE-cadherin. The BBB exerts a precise control on the exchange of substances between blood and brain tissue, protecting the brain from toxic substances (Abbott et al., 2010). Maintaining a stable microenvironment and providing oxygen, glucose and other energy substances is essential to ensure the normal function of the central nervous system (CNS) (Zhao et al., 2015). Disruptions of the BBB and secondary brain edema play vital roles in neuronal degeneration and subsequent psychiatric disorders, such as major depressive disorder, anxiety disorders, and other neurodegenerative disorders (Najjar et al., 2013). A recent study showed restraint stress could damage the BBB in the frontal cortex and hippocampus of adult rats (Santha et al., 2015). Owing to the importance of the amygdala in controlling emotion and fear memory and its high sensitivity to stress, the BBB in the amygdala may be more vulnerable to restraint stress-induced damage. However, the impact of restraint stress on the amygdalar BBB and its mechanisms remain to be elucidated.

Therefore, the present study aims to comprehensively demonstrate the impact of restraint stress on the BBB of the amygdala, including permeability, molecular biological changes, and morphological modifications.

## Materials and Methods

### Materials

Antibodies were obtained from the following sources: GLUT-1 (07-1401), Claudin-5 (ABT45) were purchased from Millipore (Temecula, CA, United States); GAPDH (ab8245), CD-31 (ab24590), Dylight-488-labeled goat anti-rabbit IgG (Abacm,ab96899) and Dylight-594-labeled goat anti-mouse IgG (Abacm,ab96873) were purchased from Abcam (San Francisco, CA, United States); AQP-4 (A5971) and GFAP (G6171) from Sigma (St Louis, MO, United States); Occludin from Thermo Fisher (Waltham, MA, United States); ZO-1 (GTX108613) from GenTex (Irvine, CA, United States); and VE-cadherin (ARG20566) from Arigo (Hsinchu, Taiwan, China).

### Animals

All experiments were performed according to the Guideline for the Care and Use of Laboratory Animals. The experimental protocols were approved by the Local Committee of Animal Care, Use and Protection of the Hebei Medical University. Male Sprague–Dawley rats (6–8 weeks of age; body weight 200–250 g) were purchased from Beijing Vital River Laboratory Animal Technology Co., Ltd. (Beijing, China); and were housed under standard laboratory conditions, with a 12-h light/12-h dark cycle, at constant temperature (22 ± 1°C) and humidity (55 ± 5%) in a conventional animal house, with standard rodent chow and tap water being freely available. These rats were adapted to the environment for at least 7 days before their use in the experiment. ELISA, EB BBB permeability, Immunohistochemistry, Western Blot and Electron Microscope analysis used a separated batch of animals. In detail, 3 rats were used for ELISA (IL-1β) analysis, 3 rats were used for EB BBB permeability analysis and 3 rats were used for Electron Microscope analysis. 9 rats were used for EPM test analysis, 3 of which were used for Immunohistochemistry analysis and 6 of which were used for Western Blot, ELISA (CORT and S100B) analysis. The total amount of animals in each group for the whole study was eighteen.

### Stress Protocol

Rats were randomly assigned to six groups: Group 1 (*n* = 18) included control animals that were handled for 5 min every day, involving transfer to a new cage and then return to the home cage. Fasting of solids and liquids began at approximately 8:00 AM each day for a duration of 6 h. Groups 2–6 (*n* = 18) were subjected to restraint stress for 6 h/day (beginning at 8:00 AM) for durations of 1, 3, 7, 14, and 21 days. Rats were exposed to only one restraint stress exposure each day. Restraint stress involved the use of a plastic tube (length 20 cm, inner diameter 5.5 cm) to block their movements.

### Body Weight Measurements

The body weight of all animals in each group was measured every morning for the duration of the experiment.

### Elevated Plus Maze (EPM) Test

The animals were tested in the EPM, 24 h after the final restraint/control handling session, to evaluate their behavioral changes. The maze consisted of two open arms and two enclosed arms. The apparatus was 50 cm from the ground. All sides and bottom surfaces of the opened and enclosed arms were constructed using black Plexiglas. Initially, each rat was gently placed in the center of the maze facing an enclosed arm and allowed to explore freely for 5 min. The time spent in the open arms, as a percentage of the total time spent exploring the EPM, was measured during each 5 min test. In addition, the numbers of entries into the open arms, as a percentage of the total number of entries, were also recorded. Finally, the percentages of time spent in open arms and entries into the open arms of total entries were used as an index of anxiety-like behavior. EPM testing was performed once per rat.

### Enzyme-Linked Immunosorbent Assay

Serum levels of corticosterone (CORT), S100B and IL-1β were measured using a CORT ELISA kit (Arigo, ARG80652, Hsinchu, Taiwan, China), a S100B ELISA kit (BioVendor, RD192090100R, Asheville, NC, United States) and an IL-1β ELISA kit (ExCell Biotech, ER008, Shanghai, China) respectively, following the manufacturer’s instructions. Animals were anesthetized by injecting 10% chloral hydrate (4 ml/kg) intraperitoneally, and blood samples were obtained by cardiac puncture. These samples were centrifuged for 5 min at 10000 × rpm and 4°C. Serum was collected in 1.5 ml EP tubes and cryopreserved at -80°C for subsequent CORT and S100B quantification.

### Evaluation of BBB Permeability

The effect of restraint stress on the permeability of the amygdalar BBB in rats was assessed by measuring Evans Blue (EB) (Promega, Beijing, China) leakage. The rats were given 4 ml/kg EB by tail intravenous injection as a 2% EB solution in physiological saline, and were anesthetized by intraperitoneal injection of 10% chloral hydrate 2 h later. The chest was opened to transcardially perfuse 200 ml of 37°C physiological saline from the left ventricle to the right atrium until colorless fluid was observed.

The rats were decapitated, and the brains were removed immediately. They were then placed on ice to bilaterally dissect the amygdala. The amygdalar tissues were weighed and homogenized in 1 ml phosphate-buffered saline (PBS). 1 ml of 60% trichloroacetic acid (TCA) was then added, and 2 min of vortex-mixing followed in order to precipitate total proteins. The homogenates were then cooled for 30 min and centrifuged for 20 min at 10000 × *g*, and 4°C. Finally, the absorbance of amygdalar supernatants for EB was measured at 610 nm by a microplate reader (Thermo MULTISKAN GO, Thermo Fisher Scientific, MA, United States). The EB content was calculated according to standard curves (ng/ml), and expressed as ng/mg of tissue protein (Hajiluian et al., 2017).

### Immunohistochemistry

All rats were deeply anesthetized after their last restraint stress procedure with intraperitoneal 10% chloral hydrate (4 ml/kg). They were then perfused transcardially with 100 ml of physiological saline (0.9% NaCl, 37°C), followed by 250 ml of cold 4% paraformaldehyde in phosphate buffered saline (PBS, 0.01 mol/L, pH = 7.4). The brains were removed immediately and post-fixed for 24 h in the same stationary liquid at room temperature. The brains were then dissected, dehydrated with increasing concentrations of ethanol, and embedded in paraffin. After completion of the embedding steps, 6 μm-thick sections were cut serially (brain sections from -1.32 to -3.48 mm posterior to bregma were analyzed), and the slices were stored at 4°C until performing immunofluorescence staining. For the immunofluorescence procedure, the slices were baked for 50 min at 60°C, then dewaxed, rehydrated and washed three times in 0.01 mol/L PBS before performing antigen retrieval by heat in a microwave oven which contained a citrate buffer. The brain slices were washed three times in PBS and incubated in endogenous peroxidase blocker solution for 30 min at room temperature. Next, non-specific binding sites were blocked with normal goat serum (ZSGB-BIO, ZLI-9056, Beijing, China) for 40 min at 37°C, and the slices were incubated at 4°C overnight with the following primary antibodies: Mouse anti-CD-31 (1:200), mouse anti-GFAP (1:200), rabbit anti-Glut-1 (1:100), rabbit anti-Claudin-5 (1:100), rabbit anti-AQP-4 (1:100), rabbit anti-Occludin (1:100), rabbit anti-ZO-1 (1:100), and rabbit anti-VE-cadherin (1:100). The following day, slices were washed three times in PBS and incubated with the corresponding secondary antibodies: Dylight-488-labeled goat anti-rabbit IgG and Dylight-594-labeled goat anti-mouse IgG for 40 min at 37°C. The slices were then washed in PBS six times for 5 min, mounted in Fluoroshield Mounting Medium with DAPI (Abcam, ab104129, San Francisco, CA, United States) and sealed with a coverslip.

Three rats from each group were used for morphological observation and data analysis. According to the stereotaxic atlas, the largest amygdala area was accurately exposed. Using the serial slice technique, we took one out of every four slices and selected a total of three slices for each rat. In each slice, four visual fields were randomly selected in the amygdala for analysis. And every image was acquired from each visual field. In total, twelve images from the amygdala area in each rat were captured using constant exposure settings by a Leica SP8 (Leica Microsystems GmbH, Wetzlar, Germany) confocal laser scanning microscope for immunohistochemistry analysis. Non-overlapping digital images (512 × 512 pixels) were taken with standardized parameter settings. Image processing was performed using Leica Application Suite X. The target proteins fluorescence intensity and the reference fluorescence intensity of each image were generated automatically by Leica Application Suite X. Then the area occupied of target proteins fluorescence intensity in each image was evaluated by calculating the ratio of target proteins fluorescence intensity/ reference fluorescence intensity. The area occupied of target proteins fluorescence intensity of each rat was evaluated by calculating the average area occupied of those twelve images. Finally, the area occupied of target proteins fluorescence intensity was expressed as target proteins /reference proteins area ratio. GFAP (Glial Fibrillary Acidic Protein) is a specific biomarker of astrocytes and CD-31 (Platelet endothelial cell adhesion molecule-1, PECAM-1/CD31) is a specific biomarker of vascular endothelial cells. GFAP and CD-31 expression are relatively constant in astrocytes and in vascular endothelial cells. And in the preliminary experiment the expression of GFAP and CD-31 in the amygdala issues was detected by Western Blot and no obvious changes were observed between the control and restraint-stressed groups. Therefore, to assess the area occupied of target proteins fluorescence intensity, the GFAP protein was used for the reference fluorescence intensity of AQP-4 and CD-31 was used for the reference fluorescence intensity of GLUT-1, TJ proteins and AJ proteins. Meanwhile, we calculated the coefficient of error (CE) according to the equation 
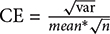
 (Gundersen and Jensen, 1987), and most of the CE in our image analysis were less than the level of 0.05. It proved the validity of the image analysis.

### Western Blot Analysis

After decapitation, the brains were removed immediately, bilateral amygdalar tissues were dissected on ice and then mechanically homogenized with a RIPA Lysis Buffer (Solarbio, R0010, Beijing, China) which included Protease Inhibitor Cocktail (Promega, 0000259656, Beijing, China). After being placed on the ice for 30 min, the lysates were centrifuged for 5 min at 14000 × *g* and 4°C. The supernatants were then collected and cryopreserved at -80°C until used. The protein concentrations were calculated with a BCA Protein Assay Kit (Multi Sciences, PQ0011, Hangzhou, China). Equal amounts of total proteins were then loaded on 6–15% polyacrylamide gels (ZomanBio, ZD304A, Beijing, China). The proteins were separated with a SDS–PAGE in Bio-Rad electrophoresis tank apparatus. Thereafter, the proteins were transferred by electrophoresis to polyvinylidene fluoride (PVDF) membranes (Bio Rad, 1620177, Berkeley, CA, United States) with a Bio-Rad *Trans*-blot turbo transfer system and blocked with 5% non-fat milk at 37°C for 1 h. The membranes were, respectively, incubated with anti-GAPDH (1:1000), anti-AQP-4 (1:500), anti-ZO-1 (1:500), anti-Claudin-5 (1:200), anti-Occludin (1:200), anti-GLUT-1 (1:1000) and anti-VE-cadherin (1:500) overnight at 4°C. After incubation with the primary antibodies, the membranes were incubated with the IRDye 680LT secondary antibodies (LI-COR, Lincoln, NE, United States) for 1 h at 37°C, away from light. Specific protein bindings were visualized with Azure C500 (Azure Biosystems, Dublin, CA, United States). The optical density of the blots were quantified using ImageJ 1.6 software (NIH, Bethesda, MD, United States).

### Transmission Electron Microscopy

For the transmission electron microscopy assays, the animals were anesthetized intraperitoneally and perfused with 150 ml of physiological saline (0.9% NaCl, 37°C), followed by phosphate buffered saline (PBS, 0.01 mol/L, pH = 7.4) containing 2% paraformaldehyde and 2.5% glutaraldehyde. After perfusion, the amygdala was removed and post-fixed with 4% glutaraldehyde for 4 h. Tissues were washed three times for 10 min with a 1/15 mol/L phosphate buffer, and post-fixed with 1% osmium tetroxide for 2 h at 4°C. Tissues were then washed again three times for 10 min with a 1/15 mol/L phosphate buffer, and dehydrated with an increasing gradient of acetone. Tissues were soused with embedding liquid and acetone, then incubated with pure resin at 37°C for 24 h, followed by 60°C for 48 h to complete the polymerization. Afterward, the samples were cut into 50 nm ultrathin slices using a Leica UC-7 ultramicrotome, and stained with uranyl acetate for 40 min as well as lead citrate for 30 min. Lastly, the slices were observed and captured with a transmission electron microscope (Hitachi H7500, Tokyo, Japan).

### Statistical Analysis

The data are presented as the means ± SD. All statistical analyses were performed with SPSS 22.0 software (IBM, Chicago, IL, United States). Statistical comparisons were analyzed using the one-way ANOVA followed by Tukey *post hoc* test for multiple comparisons. However, since the variances in all groups were not always equal, some data were analyzed with a non-parametric Kruskal–Wallis test followed by Dunn’s multiple comparison tests. Differences were considered statistically significant when the *p*-value was less than 0.05.

## Results

### Changes in Body Weight Gain and CORT Levels as Markers of Stress Response

**Figure 1:**
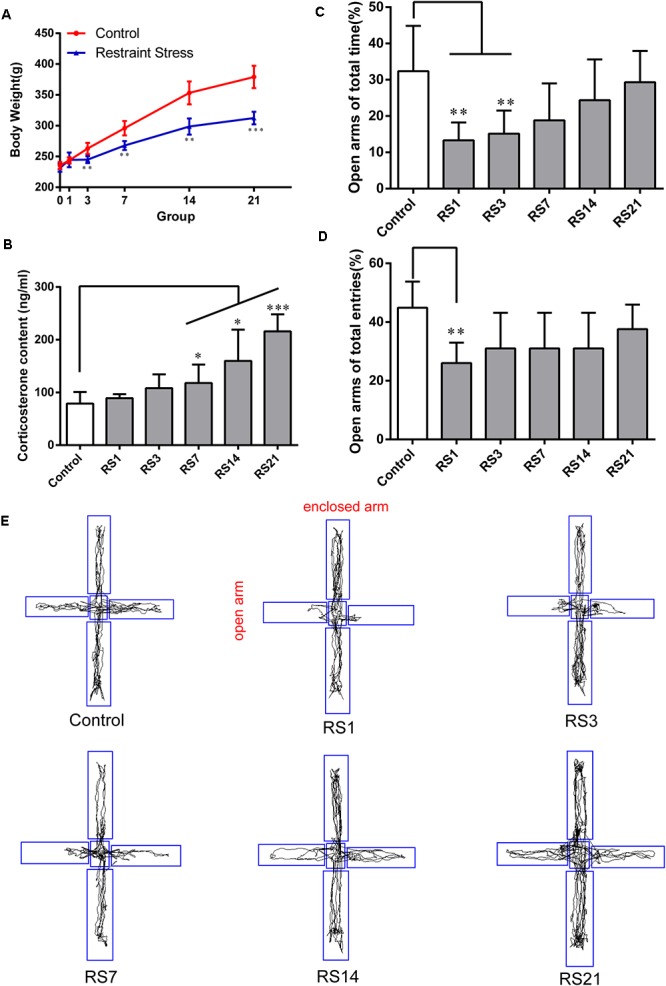
Effect of different durations of restraint stress on the body weight, serum CORT levels, and anxiety-like behavior of rats. **(A)** Restraint stress significantly reduced the body weight gain of rats from the 3rd day, compared with the control group. **(B)** Compared with the control animals, the levels of serum CORT significantly increased after stress exposure for 7 days, peaking at 21 days. **(C)** EPM ratio for Open arms time / Total time among different groups. **(D)** EPM ratio for Open arms entries / Total entries among different groups. **(E)** EPM movement track of rats in each group. Results are presented as means ± SD, *n* = 6 for Body weight and CORT analysis, *n* = 9 for EPM test analysis. Data were analyzed by One-way ANOVA followed by a Tukey *post hoc* test. ^∗^*p* < 0.05, ^∗∗^*p* < 0.01, and ^∗∗∗^*p* < 0.001 compared with the control.

Changes in body weight gain and CORT levels are considered physiological indicators of stress. To evaluate the impact of the stress model on the rats, we monitored their body weight and serum CORT levels. Compared with control animals, a slower increase in body weight was observed in restraint-stressed rats (Figure 1A). Similarly, a significant increase in serum CORT levels [*F*(5,30) = 13.518, *p* < 0.001] was detected in restraint-stressed rats in comparison with controls (78.89 ± 22.10) at 7 days (117.96 ± 34.92, *p* = 0.048), 14 days (159.95 ± 59.10, *p* = 0.018) and 21 days (215.82 ± 32.40, *p* < 0.001) (Figure 1B). Based on these parameters, we concluded that a successful restraint stress model in rats was established.

### Changes of Behavior Induced by Restraint Stress

A significant decrease in the percentage of time spent in open arms of the maze [*F*(5,48) = 5.445, *p* = 0.001] was observed for restraint-stressed rats at 1 day (13.33 ± 4.92, *p* = 0.003) and 3 days (15.14 ± 6.39, *p* = 0.008), compared with the control (32.36 ± 12.51) rats. A significant decrease in the percentage of entries into the open arms of the maze [*F*(5,48) = 4.005, *p* = 0.005] was observed for restraint-stressed rats at 1 day (26.02 ± 6.98, *p* = 0.003), compared with the control (44.85 ± 8.94) rats (Figure 1C–E). These results indicate that restraint stress induces anxiety-like behavior.

### Restraint Stress-Induced Hyperpermeability of the BBB

**Figure 2:**
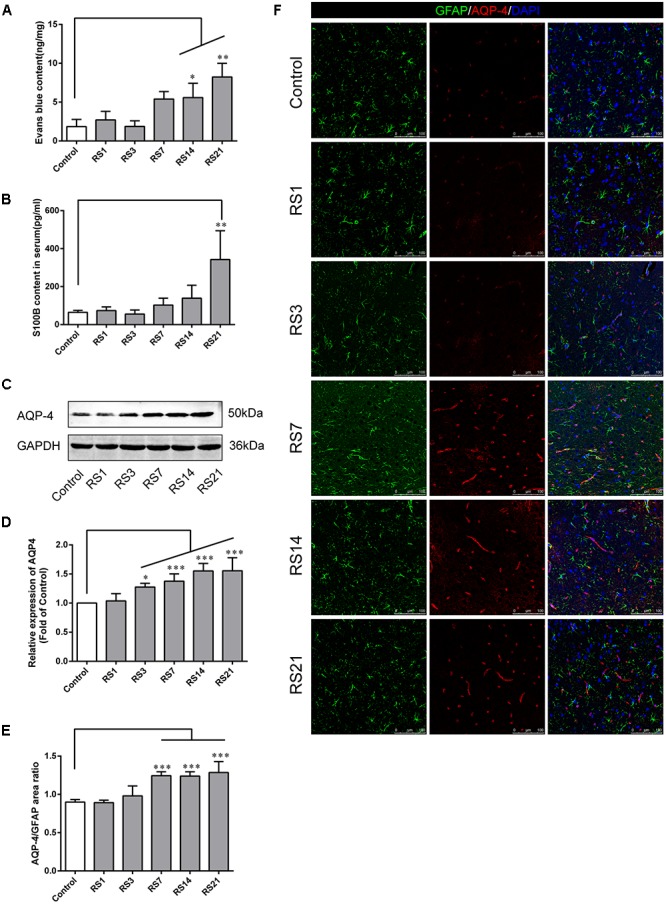
Effect of restraint stress on BBB permeability of rats. **(A)** The quantitative analysis of Evans Blue (EB) leakage in amygdala. Compared with the control, extravasation of EB in the amygdala increased significantly after stress exposure for 14 days. **(B)** The content of S100B in the serum also increased after stress exposure for 21 days. **(C,D)** Expression of AQP-4 in the amygdala of the control and stress groups by western blot analysis. **(E)** Quantitative assessment of AQP-4 positive astrocytes. **(F)** Immunofluorescence staining for AQP-4 (red) and GFAP (green) in the amygdala of the control and stress groups. Merged images of AQP-4 and GFAP staining are also shown. Results are presented as means ± SD, *n* = 3 for EB and Immunohistochemistry analysis, *n* = 6 for S100B and Western Blot analysis. Data were analyzed by One-way ANOVA followed by a Tukey *post hoc* test and a non-parametric Kruskal–Wallis test followed by Dunn’s multiple comparison tests. ^∗^*p* < 0.05, ^∗∗^*p* < 0.01, and ^∗∗∗^*p* < 0.001 compared with the control.

To appraise the effects of restraint stress on BBB permeability, we assessed EB leakage in the amygdala and serum S100B levels. Our results showed there was a significant elevation of EB [*F*(5,12) = 11.702, *p* < 0.001] leakage in rats subjected to restraint stress for 14 (5.58 ± 1.85, *p* = 0.037) and 21 (8.23 ± 1.76, *p* = 0.001) days, compared with the control (1.84 ± 0.92) rats (Figure 2A). S100B is specifically expressed in the cytoplasm and protuberances of astrocytes, and is considered a marker of BBB permeability and function. We found elevated serum S100B levels in the restraint-stressed group of 21 (342.95 ± 151.33, *p* = 0.006) days, compared with the control group (64.07 ± 10.58), further confirming increased BBB permeability (Figure 2B). These results suggest that restraint stress increases BBB permeability in the amygdala of rats.

### Increased Expression of AQP-4 by Astrocytes in the BBB Induced by Restraint Stress

AQP-4 is mainly expressed in astroglial endfeet, and is seen as a marker of BBB permeability. First, the expression of AQP-4 in the amygdala was quantified by Western Blot analysis [*F*(5,30) = 20.948, *p* < 0.001]. The results showed dramatically and significantly higher expression in the restraint-stressed groups at 3 (1.28 ± 0.06, *p* = 0.01), 7 (1.38 ± 0.13, *p* < 0.001), 14 (1.55 ± 0.13, *p* < 0.001), and 21 (1.56 ± 0.22, *p* < 0.001) days in comparison with the control rats (Figure 2C,D). Next, double-labeling immunofluorescence staining was performed to assess the fluorescence intensity ratio of AQP-4 to GFAP in the amygdala [*F*(5,12) = 27.336, *p* < 0.001]. The control rats (0.90 ± 0.03) showed moderate immunoreactivity to AQP-4 and GFAP, while restraint stress induced a significant increase of the ratio of AQP4 fluorescence intensity/GFAP fluorescence intensity, especially in the groups at 7 (1.24 ± 0.05, *p* < 0.001), 14 (1.24 ± 0.06, *p* < 0.001), and 21 (1.29 ± 0.14, *p* < 0.001) days (Figure 2E,F). The results of immunofluorescence assay was generally consistent with the Western Blot analysis.

### Decreased Expression of GLUT-1 Induced by Restraint Stress

**Figure 3:**
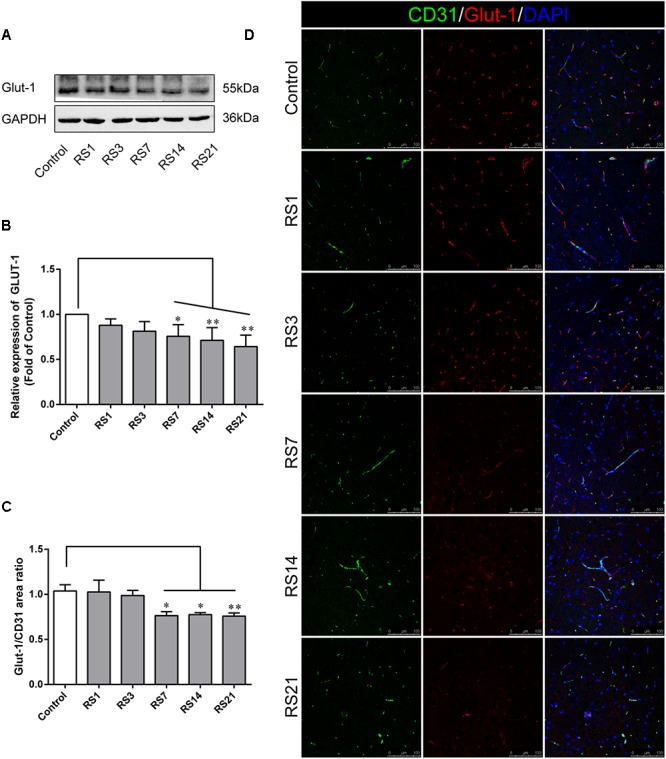
Effect of restraint stress on the expression of GLUT-1 in the amygdala. **(A)** Representative blots of GLUT-1 by western blot. **(B)** Quantitative data of Glut-1. **(C)** Quantitative assessment of GLUT-1 positive blood vessels. **(D)** Immunofluorescence staining for Glut-1 (red) and CD31 (green) in the amygdala of the control and stress groups. Merged images of i1 and CD31 staining are also shown. Results are presented as means ± SD, *n* = 3 for Immunohistochemistry analysis, *n* = 6 for Western Blot analysis. Data were analyzed by a non-parametric Kruskal–Wallis test followed by Dunn’s multiple comparison tests. ^∗^*p* < 0.05 and ^∗∗^*p* < 0.01 compared with the control.

Glucose transporter-1 (GLUT-1) is the only glucose transporter expressed in the BBB. Expression of GLUT-1 has been reported to play a vital role in the development of brain microvasculature and maintaining BBB integrity, and it appears to be decreased when the BBB is structurally damaged. The expression of GLUT-1 was quantified by Western Blot analysis. Results showed a significant down-regulation in the restraint- stressed rats at 7 (0.76 ± 0.13, *p* = 0.03), 14 (0.71 ± 0.14, *p* = 0.004), and 21 (0.64 ± 0.13, *p* = 0.001) days, compared with the control rats (Figure 3A,B). The fluorescence intensity ratio of GLUT-1 to CD31 was further assessed by double-labeling immunofluorescence staining. Compared with the control rats (1.04 ± 0.07), the results indicated decreased ratio of GLUT-1 fluorescence intensity/CD-31 fluorescence intensity in the restraint-stressed rats at 7 (0.77 ± 0.04, *p* = 0.014), 14 (0.78 ± 0.02, *p* = 0.027), and 21 (0.76 ± 0.04, *p* = 0.007) days (Figure 3C,D).

### Down-Regulation of Tight Junction and Adherens Junction Proteins Induced by Restraint Stress

**Figure 4:**
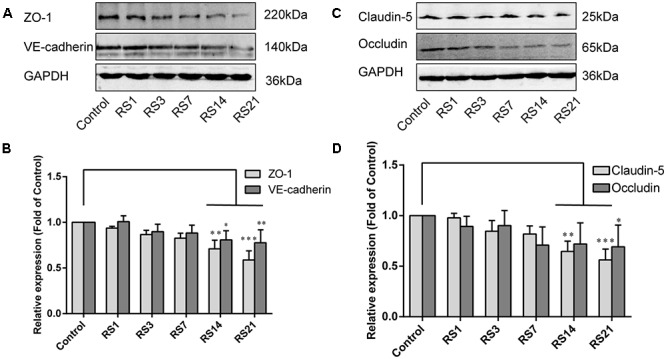
Effect of restraint stress on the expression of TJ proteins and AJ proteins in the amygdala by western blot analysis. **(A)** Representative blots of ZO-1 and VE-cadherin. **(B)** Quantitative data of ZO-1 and VE-cadherin. **(C)** Representative blots of Claudin-5 and Occludin. **(D)** Quantitative data of Claudin-5 and Occludin. Results are presented as means ± SD, *n* = 6. Data were analyzed by a non-parametric Kruskal–Wallis test followed by Dunn’s multiple comparison tests. ^∗^*p* < 0.05, ^∗∗^*p* < 0.01, and ^∗∗∗^*p* < 0.001 compared with the control.

**Figure 5:**
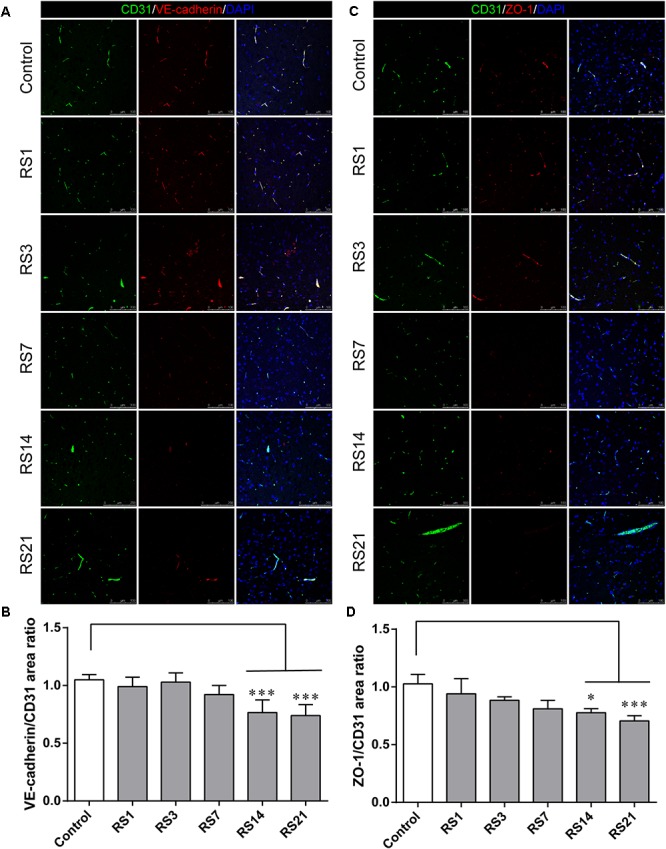
Immunofluorescence intensity analysis (area ratio of fluorescence intensity) of TJ and AJ in amygdala of rats following restraint stress. **(A)** Immunofluorescence staining for VE-cadherin (red) and CD31 (green) in the amygdala of the control and stress groups. Merged images of VE-cadherin and CD31 staining are also shown. **(B)** Quantitative assessment of VE-cadherin positive blood vessels. **(C)** Immunofluorescence staining for ZO-1 (red) and CD31 (green) in the amygdala of the control and stress groups. Merged images of ZO-1 and CD31 staining are also shown. **(D)** Quantitative assessment of ZO-1 positive blood vessels. Results are presented as means ± SD, *n* = 3. Data were analyzed by One-way ANOVA followed by a Tukey *post hoc* test and a non-parametric Kruskal–Wallis test followed by Dunn’s multiple comparison tests. ^∗^*p* < 0.05 and ^∗∗∗^*p* < 0.001 compared with the control.

**Figure 6:**
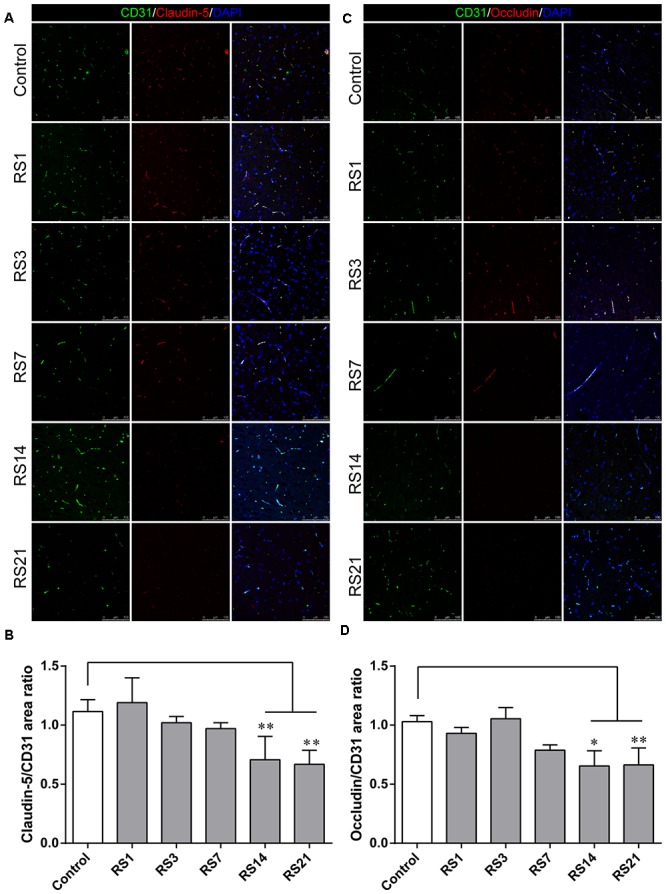
Immunofluorescence intensity analysis (area ratio of fluorescence intensity) of TJ in amygdala of rats following restraint stress. **(A)** Immunofluorescence staining for Claudin-5 (red) and CD31 (green) in the amygdala of the control and stress groups. Merged images of Claudin-5 and CD31 staining are also shown. **(B)** Quantitative assessment of Claudin-5 positive blood vessels. **(C)** Immunofluorescence staining for Occludin (red) and CD31 (green) in the amygdala of the control and stress groups. Merged images of Occludin and CD31 staining are also shown. **(D)** Quantitative assessment of Occludin positive blood vessels. Results are presented as means ± SD, *n* = 3. Data were analyzed by a non-parametric Kruskal–Wallis test followed by Dunn’s multiple comparison tests. ^∗^*p* < 0.05 and ^∗∗^*p* < 0.01 compared with the control.

Both TJ and AJ are important components of the BBB neurovascular unit, sustaining its integrity and normal function. In order to determine the effects of restraint stress on TJ and AJ proteins, Western Blot analysis was performed to quantify the expression of TJ proteins (Claudin-5, Occludin, ZO-1) and a prominent AJ protein (VE-cadherin). In addition, the immunostaining intensity of TJ proteins (Claudin-5, Occludin, ZO-1) and the AJ protein (VE-cadherin) were analyzed with Leica TCS SP8 software. The fluorescence intensity ratios of Claudin-5 to CD31, Occludin to CD31, ZO-1 to CD31 and VE-cadherin to CD31 were measured. Western Blot results showed significantly reduced expressions of Claudin-5, ZO-1, and VE-cadherin in the restraint-stressed rats at 14 (Claudin-5: 0.65 ± 0.10, *p* = 0.002, ZO-1:0.71 ± 0.09, *p* = 0.001, VE-cadherin: 0.81 ± 0.10, *p* = 0.01) and 21 (Claudin-5: 0.56 ± 0.11, *p* < 0.001, ZO-1: 0.59 ± 0.10, *p* < 0.001, VE-cadherin: 0.78 ± 0.14, *p* = 0.005) days, along with a down-regulation of occludin in rats at 21 (0.69 ± 0.22, *p* = 0.037) days, in comparison with the control group (Figure 4). The immunofluorescence staining results suggested that there were significant decreases of the ratios of Claudin-5, ZO-1, VE-cadherin [*F*(5,12) = 15.204, *p* < 0.001] and Occludin fluorescence intensity/CD-31 fluorescence intensity in the restraint-stressed rats at 14 (Claudin-5: 0.71 ± 0.20, *p* = 0.009, ZO-1: 0.78 ± 0.03, *p* = 0.01, VE-cadherin: 0.77 ± 0.11, *p* < 0.001, Occludin: 0.65 ± 0.13, *p* = 0.011) and 21 (Claudin-5: 0.67 ± 0.12, *p* = 0.003, ZO-1: 0.71 ± 0.04, *p* < 0.001, VE-cadherin: 0.74 ± 0.10, *p* < 0.001, Occludin: 0.66 ± 0.14, *p* = 0.009) days, compared with the control rats (Claudin-5: 1.12 ± 0.10, ZO-1: 1.03 ± 0.08, VE-cadherin: 1.05 ± 0.05, Occludin: 1.03 ± 0.05) (Figure 5, 6). Taken together, these findings indicate restraint stress could lead to damage and dysfunction of the BBB.

### Transmission Electron Microscopy of BBB

**Figure 7:**
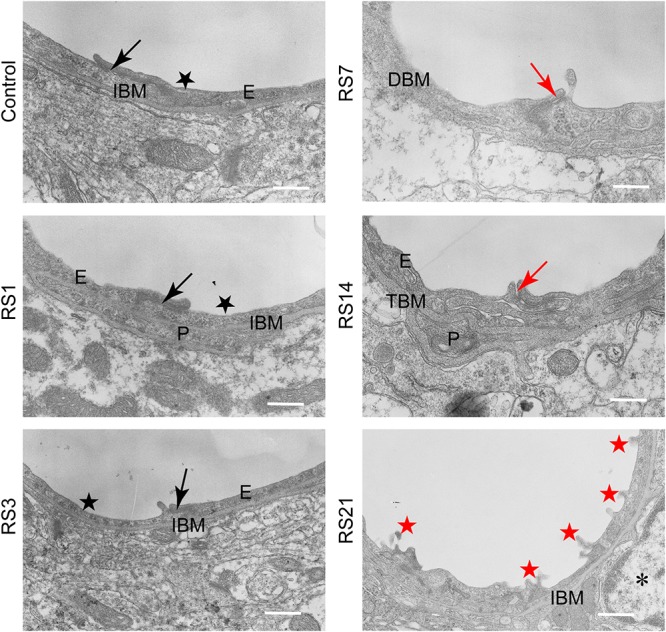
Transmission Electron Microscopy graphs of BBB in the amygdala of control and stressed rats. E, brain microvascular endothelial cell; P, pericyte; IBM, intact basal membrane; DBM, dissolved basal membrane; TBM, thickened basal membrane; TJ, tight junction; black arrows (→) indicate intact tight junctions; red arrows (→) indicate discontinuous or open tight junctions; black pentagrams (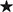) point to smooth luminal membrane; red pentagrams (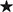) point to luminal membrane protrusions; asterisk marks edematous astroglial endfeet. Scale bar: 500 nm, *n* = 3.

Transmission electron microscopy revealed ultrastructural changes in the amygdalar capillaries. In the control rats, the capillary luminal membranes were smooth, the basal membranes were intact and the TJ between BMVEC presented thick electron density, indicating complete structure of the BBB. Morphological deformities were observed in the BMVEC of rats at 7 days of stress. The basal membranes were partly dissolved and the number of open TJ were increased. Restraint stress for 14 days resulted in more obvious ultrastructural changes in TJ, basal membranes and brain capillary lumina. Representative electron microscopy images showed that the space in TJ was increased, the basal membranes were thickened and the capillary luminal membranes were rough. In rats subjected to restraint stress for 21 days, intraluminal protrusions of brain capillaries and partial detachments of endothelial cells were observed, along with edematous astroglial endfeet and discontinuous or open TJ (Figure 7 and Table 1).

**Table 1:** Summary of changes in amygdala BBB ultrastructure following restraint stress in adult rats.

			Control	RS1	RS3	RS7	RS14	RS21
Number of capillaries			19	18	15	17	15	12
Number of images			23	23	16	22	18	15
Brain capillary endothelial cell	Luminal membrane	Smooth	95%	94%	93%	82%	80%	75%
		Protrusions	5%	6%	7%	18%	20%	25%
	Tight junctions	Intact	95%	95%	94%	81%	67%	73%
		Discontinuous	5%	5%	6%	19%	33%	27%
	Basal membrane	Intact	100%	94%	100%	76%	73%	92%
		Increased thickness	0%	0%	0%	6%	13%	8%
		Dissolution	0%	0%	0%	12%	7%	0%
		Detachment	0%	6%	0%	6%	7%	0%
Astroglia endfeet		Intact	100%	100%	100%	94%	93%	83%
		Edematous	0%	0%	0%	6%	7%	17%
**Ultrastructural alterations of the BBB following restraint stress in rats**		
TJ integrity				—	—	*↓*	*↓*	↓
BM integrity				↓	—	*↓*	*↓*	***↓***
Astroglia edema				—	—	*+*	*+*	*+*

*Values are shown as percent of all images analyzed in the group, except for tight junctions, which are given as percent of all junctions observed in the group. BM, basal membrane; TJ, tight junction; —, no change; ↓, decrease; +, edema observed*.

### Increased Level of IL-1β Induced by Restraint Stress

**Figure 8:**
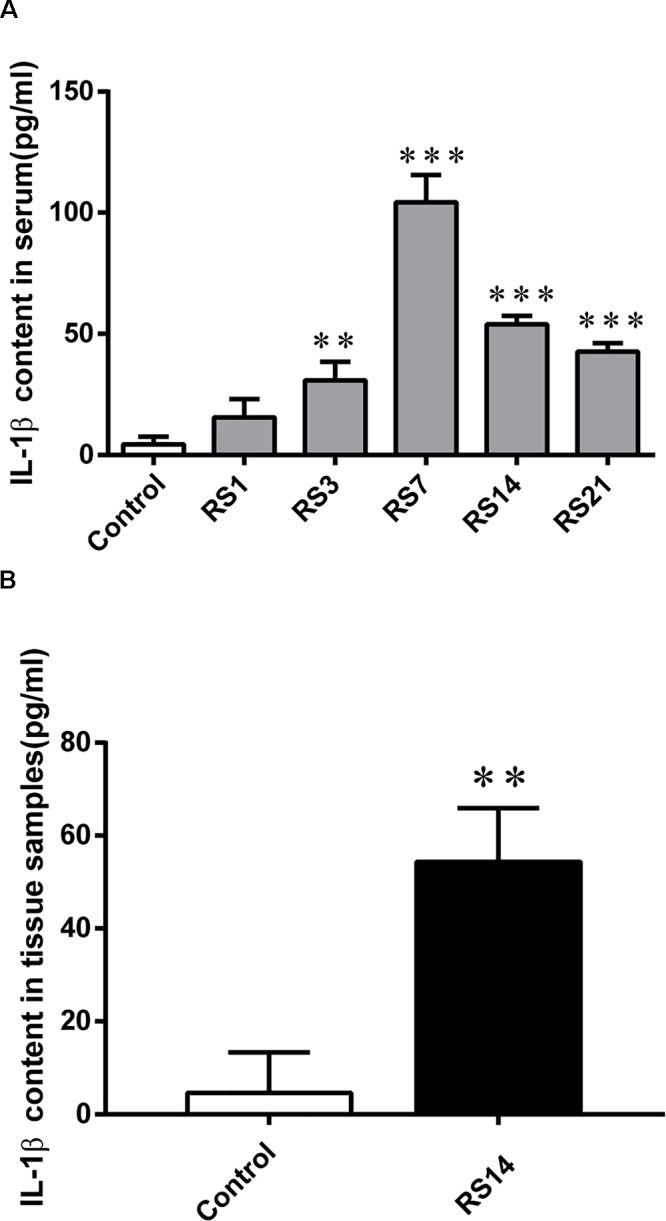
IL-1β content in serum and amygdalar tissues. **(A)** A significant increase was detected in serum IL-1β levels in restraint-stressed rats in comparison with the controls at days 3, 7, 14, and 21, peaking at 7 days. **(B)** IL-1β content in amygdalar tissues was increased by restraint stress. Results are presented as means ± SD, *n* = 3. Data were analyzed by One-way ANOVA followed by a Tukey *post hoc* test. ^∗∗^*p* < 0.01 and ^∗∗∗^*p* < 0.001 compared with the control.

Numerous studies have reported that inflammatory cytokines, such as IL-1β, could induce BBB disruption. Our results revealed a significant increase in serum IL-1β levels [*F*(5,12) = 81.224, *p* < 0.001] in restraint-stressed rats in comparison with the controls (4.38 ± 3.18) at days 3 (30.82 ± 7.61, *p* = 0.005), 7 (104.28 ± 11.27, *p* < 0.001), 14 (53.89 ± 3.56, *p* < 0.001), and 21 (42.63 ± 3.55, *p* < 0.001), peaking at 7 days (Figure 8A). Similarly, a significant increase in amygdalar tissue IL-1β levels [*F*(1,14) = 35.316, *p* = 0.004] was detected in restraint-stressed rats (54.35 ± 11.57, *p* = 0.001), compared with the controls (4.63 ± 8.72, Figure 8B).

## Discussion

The BBB is not only a natural barrier between the CNS and the peripheral system, it also plays a vital role in maintaining the neuronal microenvironment and brain homoeostasis (Liu et al., 2017). Disruption of the BBB can lead to brain edema, accumulation of toxic substances in the CNS, and induce psychiatric disorders (Najjar et al., 2013). Numerous studies have shown that a variety of factors can cause disruptions of the BBB (Zhang et al., 2012; Nair et al., 2015; Gao et al., 2017; Perez-Hernandez et al., 2017; Qie et al., 2017; Bennett et al., 2018). Few studies have focused on the underlying harmful effects of restraint stress on the BBB. Previous studies have reported immobilization stress increased the permeability of the BBB, mainly in younger rats (Sharma and Dey, 1981). However, another study reported acute swim and restraint stress did not increase BBB permeability in mice (Roszkowski and Bohacek, 2016). Recent research revealed restraint stress could induce BBB changes in adult rats, but the study only focused on BBB morphological changes in the frontal cortex and hippocampus (Santha et al., 2015). In summary, these findings related to stress-induced BBB alterations are very limited and remain controversial. Compelling evidence has demonstrated that stress raises serum CORT levels by activating the hypothalamus-pituitary-adrenal axis (Conrad, 2010; Markovic et al., 2011). Furthermore, reduced weight gain and increased CORT serum levels are viewed as markers for stress severity (Garabadu et al., 2011; Iniguez et al., 2014). The EPM test is viewed as a method to accurately verify the effectiveness of emotion changes following restraint stress. Our results suggest the restraint stress model was established successfully, proving its usefulness for subsequent experimental research.

We measured BBB permeability with EB, which can bind rapidly and tightly to plasma albumin via disrupted BBB (Saunders et al., 2015), and has been acknowledged as an indicator of BBB permeability in many studies (Hajiluian et al., 2017; Qie et al., 2017). In this study, the EB content in the amygdala was detected after 2 h of circulation. Following restraint stress for 14 and 21 days, the content of EB in the amygdalar homogenates was significantly elevated. We also determined serum S100B levels. S100B is a calcium-binding peptide produced mainly by astrocytes (Rothermundt et al., 2003). Recently, emerging evidence has suggested S100B could be a new and non-invasive marker of BBB function and brain lesions (Kanner et al., 2003; Koh and Lee, 2014; Li et al., 2014; Wu et al., 2016). In this study, serum S100B gradually increased after restraint stress for 21 days, which indicated increased permeability, compared with the controls. These results are in line with most previous research.

In addition to BMVEC, astrocytes also play a key role in maintaining the integrity and normal function of the BBB (Zlokovic, 2008), as astroglial endfeets are in direct contact with the BMVEC (Abbott et al., 2006). Aquaporin-4 (AQP-4) is a water channel protein chiefly expressed in the astroglial endfeets, and the protein controls water transport at the blood–brain interface. It is known to play a critical role in BBB integrity, and increased AQP-4 expression has been linked to various types of cerebral edema (Papadopoulos and Verkman, 2007; Haj-Yasein et al., 2011). Moreover, increased AQP-4 expression has been reported in the context of disturbed BBB homeostasis, and could be seen as a specific marker of BBB permeability (Liu et al., 2017). Notably, in our study, a significant increase of AQP-4 was observed in the restraint-stressed rats. Therefore, our results indicate that restraint stress could increase BBB permeability in the amygdala and induce disruption of BBB homeostasis.

The physical barrier properties of the BBB are mainly dependent on specialized cellular junctions, which include TJ complexes and AJ complexes. TJ complexes include a variety of transmembrane proteins, and are distributed between adjacent endothelial cells to limit paracellular permeability (Zhao et al., 2015). Occludin, claudin-5 and ZO-1 are the three most important TJ proteins. Down-regulation of TJ proteins can alter the cytoskeletal structure of BMVEC and induce an increase in BBB permeability (Bae et al., 2015). Decreased expression of TJ proteins has been considered an indicator of BBB structural damage (Kuan et al., 2016; Wang et al., 2016). The present study found restraint stress significantly decreased the expressions of occludin, claudin-5 and ZO-1, suggesting deterioration of BBB structure. Previous studies have mainly focused on these three important TJ proteins (Liu et al., 2012), but few studies have been conducted on AJ proteins. Increasing evidence has shown AJ proteins are essential for maintaining TJ functionality via VE-cadherin-mediated up-regulation of claudin-5 (Taddei et al., 2008). VE-cadherin is the main component of AJ, playing a vital role in the establishment, maturity and maintenance of endothelial intercellular junctions (Gavard and Gutkind, 2008; Li et al., 2018). Our study showed that VE-cadherin expression decreased after restraint stress. In the present study, restraint stress significantly decreased the expression of TJ and AJ proteins in different degrees, indicating damage to the critical structure and function of BBB in the amygdala.

Additionally, glucose is the most important energy source for the brain, and it is mainly transported across the BBB by GLUT-1, which is specifically expressed in the BBB. Previous research has reported GLUT-1 plays an important role in the development of the BBB (Zheng et al., 2010). The present study showed the expression of GLUT-1 was also decreased after exposure to restraint stress, indicating impairment of energy transport in the BBB. Furthermore, ultrastructural findings showed detached endothelial cells, defective tight junctions, edematous astroglial endfeet, and malformed capillary lumen in the restraint-stressed animals, suggesting restraint stress could induce morphological changes of the BBB in the amygdala.

Numerous studies have reported inflammatory cytokines, such as IL-1β, could induce BBB disruption (Didier et al., 2003; Simi et al., 2007; Rigor et al., 2012). To investigate the mechanism of restraint stress induced damage of the BBB, we detected the content of IL-1β in serum and amygdalar tissues. The present study showed significant high levels of the IL-1β in restraint-stressed rats both in serum and amygdalar tissues, indicating restraint stress could induce inflammatory reaction. Our results have shown restraint stress could induce damage of the BBB in the amygdala. However, it is unclear that the IL-1β detected in amygdalar tissues was produced by the amygdala itself or resulted from peripheral blood. Exploring the underlying mechanism of IL-1β induced BBB damage is a focus of our future work.

Although the report of Santha et al. (2015) explored the effect of restraint stress on the BBB, there were some differences between the report of Santha and the present study. First, hippocampus and frontal cortex, not the amygdala, were detected in Santha’s study. Second, no behavioral measurement was conducted in the previous study, however, the CORT and EPM were measured to evaluate the effectiveness of the stress model in the present study. Third, Santha’s report just provided three restraint-stressed groups (1, 3, and 21 days), while our study provided five restraint-stressed groups (1, 3, 7, 14, and 21 days),which was more conducive to the exploration of the effect of restraint stress on the BBB. Furthermore, the previous study only focused on the BBB morphological changes without quantitative analysis at molecular biological level. However, the present study comprehensively demonstrated the impact of restraint stress on the BBB of the amygdala in many respects. Not only the BBB morphological alteration, but also the changes of BBB permeability and molecular biological quantitative analysis were all detected in the present study. Interestingly, Santha’s study showed that the morphological changes of BBB began since 1 day of restraint stress, particularly TJ became discontinuous in the frontal cortex and the hippocampus. But the morphological changes in the frontal cortex were softened at 21 days of restraint stress, which might be due to a positive adaptation of the rats to restraint stress, while in the hippocampus the number of open or discontinuous junctions was still higher. However, the present study found that the structural changes and functional changes of BBB in the amygdala appeared after 7 days of restraint stress, and strengthened after 14 and 21 days of restraint stress. The discrepancy between the two papers may be due to the following reasons. First, different brain areas may present different alterations when suffer from stress. Therefore, the amygdala detected in the present study and the hippocampus and frontal cortex detected in Santha’s study may not change parallelly in pathologies. Second, SD rats (250 g) and Wistar (350 g) rats were used, respectively, in the present study and Santha’s study. So the injury effects on the BBB induced by restraint stress in our present study may be differ from Santha’s study, owing to the difference of rat species and weight.

Indeed, a limitation of our study is that there were no female control rats. In many studies, women/females are still a neglected group despite the increasing awareness of sex differences in reactivity to drugs and other treatments. The difference in the damage effects of restraint stress between male rats and female rats could not be confirmed in the present study. Therefore, further investigations are required to clarify these scientific issues.

## Conclusion

In conclusion, the present study detected significant changes on the BBB in the amygdala of rats that underwent restraint stress by multiple methods, demonstrating restraint stress could lead to the dysfunction and destruction of the BBB. The harmful effects induced by stress on the amygdalar BBB not only explain the underlying mechanisms of cognitive impairment, PTSD, depression and other neurodegenerative diseases related to stress, but also provide new targets for the treatment of these diseases.

## Author Contributions

BC, XZ, CM, YL, SL, and GX conceived this work. GX and XZ wrote the main manuscript text. GX, CW, and ZS performed the lab experiments. GX analyzed and interpreted the data. YS and LL contributed materials. All authors read and commented on the manuscript.

## Conflict of Interest Statement

The authors declare that the research was conducted in the absence of any commercial or financial relationships that could be construed as a potential conflict of interest.
